# Supplementary material for: Causal Relationship between Aging and Anorexia Nervosa: A White-Matter-Microstructure-Mediated Mendelian Randomization Analysis
Source: Biomedicines. 2024 Aug 16;12(8):1874. doi: 10.3390/biomedicines12081874 (PMC11351342; doi:10.3390/biomedicines12081874)
Supplement: Supplementary file 1 [file biomedicines-12-01874-s001.zip › Supplementary Figure/Figure S4/Singe SNP plot of MR analysis for TL to ubm-b-1710.pdf]

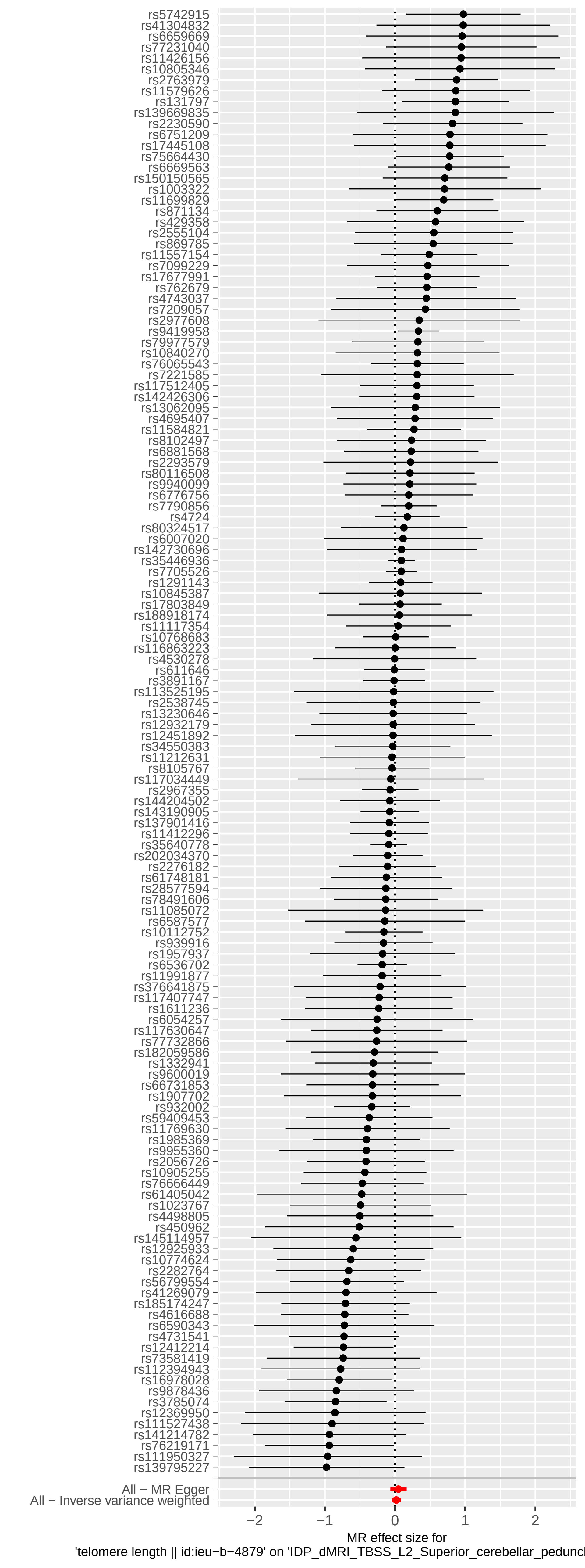
rs5742915
rs41304832
rs6659669
rs77231040
rs11426156
rs10805346
rs2763979
rs11579626
rs131797
rs139669835
rs2230590
rs6751209
rs17445108
rs75664430
rs6669563
rs150150565
rs1003322
rs11699829
rs871134
rs429358
rs2555104
rs869785
rs11557154
rs7099229
rs17677991
rs762679
rs4743037
rs7209057
rs2977608
rs9419958
rs79977579
rs10840270
rs76065543
rs7221585
rs117512405
rs142426306
rs13062095
rs4695407
rs11584821
rs8102497
rs6881568
rs2293579
rs80116508
rs9940099
rs6776756
rs7790856
rs4724
rs80324517
rs6007020
rs142730696
rs35446936
rs7705526
rs1291143
rs10845387
rs17803849
rs188918174
rs11117354
rs10768683
rs116863223
rs4530278
rs611646
rs3891167
rs113525195
rs2538745
rs13230646
rs12932179
rs12451892
rs34550383
rs11212631
rs8105767
rs117034449
rs2967355
rs144204502
rs143190905
rs137901416
rs11412296
rs35640778
rs202034370
rs2276182
rs61748181
rs28577594
rs78491606
rs11085072
rs6587577
rs10112752
rs939916
rs1957937
rs6536702
rs11991877
rs376641875
rs117407747
rs1611236
rs6054257
rs117630647
rs77732866
rs182059586
rs1332941
rs9600019
rs66731853
rs1907702
rs932002
rs59409453
rs11769630
rs1985369
rs9955360
rs2056726
rs10905255
rs76666449
rs61405042
rs1023767
rs4498805
rs450962
rs145114957
rs12925933
rs10774624
rs2282764
rs56799554
rs41269079
rs185174247
rs4616688
rs6590343
rs4731541
rs12412214
rs73581419
rs112394943
rs16978028
rs9878436
rs3785074
rs12369950
rs111527438
rs141214782
rs76219171
rs111950327
rs139795227
All − MR Egger
All − Inverse variance weighted
−2
−1
0
1
2
MR effect size for
'telomere length || id:ieu−b−4879' on 'IDP_dMRI_TBSS_L2_Superior_cerebellar_peduncl
